# Resting CD4 regulatory T cell and neuroblastoma: A Mendelian randomization study

**DOI:** 10.1002/pdi3.85

**Published:** 2024-06-08

**Authors:** RuiZong Wang, Shan Wang

**Affiliations:** ^1^ Department of Pediatric Surgical Oncology Children's Hospital of Chongqing Medical University Chongqing China; ^2^ National Clinical Research Center for Child Health and Disorders Ministry of Education Key Laboratory of Child Development and Disorders Chongqing Key Laboratory of Pediatrics Chongqing China

**Keywords:** Mendelian randomization, neuroblastoma, resting CD4 regulatory T cell

## Abstract

Neuroblastoma (NB) is a common extracranial solid tumor in children, and currently our understanding of the molecular mechanisms underlying tumor progression is not very thorough. In clinical practice, although the prognosis and survival rates of NB patients in the low‐risk and medium‐risk groups are still acceptable, the prognosis and survival rates of NB patients in the high‐risk group are extremely poor. Therefore, improving awareness of NB tumors is crucial for improving the treatment status of NB patients in clinical practice. So we collected common tumor exposure factors and performed multiple Mendelian randomization (MR) analysis on NB, and ultimately determined that the increase in Resting CD4 regulatory T cell was positively correlated with the occurrence and development of NB. In addition, we also used the network pharmacology algorithm proximity to screen the NB chemotherapy drug papain and reasonably speculated that there is an interaction between papain and CD4 regulatory T cell in the chemotherapy of NB patients.

## Graphical abstract

The increase of Resting CD4 regulatory T cell is positively correlated with the occurrence and development of neuroblastoma (NB), while the drug papain targets CD4 + T lymphocytes and has a chemotherapy effect on NB.

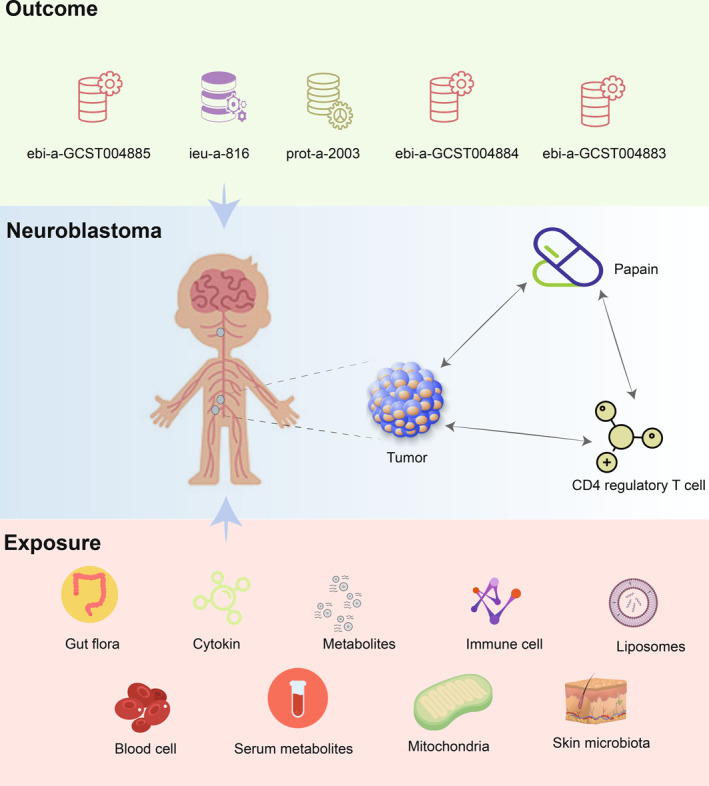

## 1 INTRODUCTION

Neuroblastoma (NB) is a highly heterogeneous extracranial solid tumor in children, originating from embryonic tumors formed by the abnormal development of sympathetic crest cells. Tumors sprout from sympathetic nerve cells from the neural crest during the fetal or early postnatal period of the child, forming in the pathway of neural crest cells migrating to the adrenal gland and sympathetic nerve chain. The adrenal gland is the most common primary site of NB, accounting for approximately 50%. Moreover, according to the degree of differentiation and development of cells, they can be further subdivided into NB, Ganglioneuroblastoma, and Ganglioneuroma (GN), collectively referred as peripheral neuroblastic tumors.^1^ Among them, NB, as a representative tumor in children, has an estimated incidence rate of 1.2 cases per 100,000 people.^2^ The Children's Oncology Group (COG) risk classification system in the United States is widely used internationally to distinguish different levels of risk in children with NB. Low‐risk children are generally treated with surgical resection, with a good prognosis and a survival rate of over 90%. On the contrary, high‐risk children usually require a combination of chemotherapy and radiation therapy, and still exhibit resistance to treatment and a tendency to relapse. The survival rate of NB children is usually less than 50%.^3^, ^4^, ^5^ Therefore, exploring the changes in molecular mechanisms associated with the onset of NB can help us better guide clinical treatment with high survival rates.

Gut microbiota is a collective term for various bacteria in the human gastrointestinal tract. It is a widely distributed, complex, and diverse microbial community that can maintain the normal physiological and immune functions of the host's intestine. The gut microbiota is causally related to cancer.^6^ In blood cells, there are reports that red blood cells can be used for high‐performance capture and release of circulating tumor cells, and can affect the tumor microenvironment through immunity.^7^, ^8^ Other blood cells can also have an impact on circulating tumor cells.^9^ Moreover, there is a complex and intricate relationship between inflammatory factors and tumors. Among them, inflammatory cells can release chemical substances mainly composed of reactive oxygen species, which can actively induce surrounding cancer cells and accelerate their transformation to a highly malignant tumor state. Interestingly, both pro‐tumor immune cells and anti‐tumor immune cells coexist, and the balance of immune responses within tumor cells also conflicts with each other.^10^ Metabolites derived from the microbiome can counteract tumor‐induced immunosuppression and enhance immune checkpoint blockade.^11^ At the same time, tumor microenvironment metabolites can guide T cell differentiation and function.^12^ In addition, research suggests that tumor metabolism and related serum metabolites are associated with cancer prognosis.^13^ Intramitochondrial electron flow can enhance tumor immunogenicity, while mitochondrial rupture can limit Natural Killer cell‐based tumor immune monitoring.^14^, ^15^ Immune cells, as a major "hotspot" in the field of tumor research, are involved in improving the infiltration of immune cells or increasing their anti‐tumor activity.^16^, ^17^, ^18^ All have demonstrated the close relationship between immune cells and tumors. Liposomes, as a common component structure of tumors, have been reported to be related to the tumor suppressor CDKN2A. Some liposomes can also maintain the occurrence of inflammation caused by tumors.^19^, ^20^ Even though skin microbiota is a less studied tumor risk factor, studies have shown that leukemia patients often carry gram‐negative bacteria on their skin.^21^ Based on all the risk factors associated with the occurrence and development of tumors mentioned above, we have reason to suspect their relationship with NB.

Due to early Mendelian randomization (MR) studies typically focusing on small sample populations and using only a small amount of genetic variation, the effectiveness of MR studies has declined. In recent years, with the release of hundreds of thousands of aggregated data on the relationship between exposure and disease and genetic variation through genome‐wide association studies (GWAS).^22^ This has led to a rapid increase in the number of MR studies. MR uses genetic variation as instrumental variables (IVs) to estimate the causal relationship between the exposure factors of interest and the outcomes of interest, where "exposure factors" refer to assumed causal risk factors, also known as intermediate phenotypes, which can be biomarkers, physical measurements, or any risk factors that may affect resolution. Generally, we list diseases as outcomes, but not limited to a specific disease.^23^ Therefore, using MR to explore the potential causal relationship between NB and exposure factors can help us better understand the molecular mechanism of NB.

Firstly, we used the gut microbiota, blood cells, immune cells, inflammatory factors, metabolites, serum metabolites, mitochondria, liposomes, and skin microbiota as the exposure dataset, and NB as the outcome dataset for the initial screening of relevant exposures. After analyzing each result, we determined the exposure of resting CD4 regulatory T cells and NB as research subjects and tested the reliability of the results through two sample MR analysis. Then, we identified NB set of genes as the underlying data for targeted drug screening through co‐localization analysis and summary‐data‐based Mendelian randomization (SMR) analysis.

## 2 MATERIALS AND METHODS

### 2.1 Mendelian randomization assumptions and data sources

According to the core idea of MR, we first need to ensure the independence of genetic variation from any confounding factors in the exposure outcome association, while ensuring the correlation between genetic variation and exposure variables, and that genetic variation is not related to outcomes through any means other than exposure factors to satisfy the hypothesis of exclusivity. Therefore, we carried out corresponding measures, heterogeneity tests, test for directional horizontal pleiotropy, and test that the exposure is upstream of the outcome.

In this study, NB was used as the outcome, so we used the keyword "neuroblastoma" on the Integrative Epidemiology Unit (IEU) Open GWAS website(https://gwas.mrcieu.ac.uk/) to conduct a search on the outcome dataset, and ultimately included all five search results from "prot‐a‐2003", "ieu‐a‐816", "ebi‐a‐GCST004883", "ebi‐a‐GCST004884", and "ebi‐a‐GCST004885" in the outcome. Among them, prot‐a‐2003 has the largest sample size, 10,534,735 SNPs, and is characterized by NB suppressor of tumorigenicity 1. The number of SNPs in ebi‐a‐GCST004883, ebi‐a‐GCST004884, and ebi‐a‐GCST004885 datasets is 504,798, corresponding to the amplification of chromosome 11q deletion, 1p deletion, and MYCN, respectively. The number of SNPs in ieu‐a‐816 is 468,788 with no prominent feature records.

We exposed the gut microbiota, blood cells, immune cells, inflammatory factors, metabolites, serum metabolites, mitochondria, liposomes, and skin microbiota. The exposure dataset downloaded from the literature was processed with default parameters (clump *r* ^2^ < 0.001, clump kb < 10,000, *p* < 5 × 10^−6^). Among them, "gut 418" is an upgraded version of "gut 211", both from the IEU Open GWAS website, while "gut 412" is the gut microbiota collected from the Dutch microbiome project, which complements each other and is not completely duplicated. "Immune cell original" is downloaded from the IEU Open GWAS website based on the keyword "Immune cell", while "Immune cell default" is from the GWAS catalog, and there is no duplication between them.

Since the data we used is based on published research and public databases, there is no need to obtain additional ethical approval from the institutional review committee. The data used in this study is summarized in the Table 1.

**TABLE 1:** Data source.

Data	From
Ieu‐a‐816	(https://gwas.mrcieu.ac.uk/)
Prot‐a‐2003	(https://gwas.mrcieu.ac.uk/)
Ebi‐a‐GCST004883	(https://gwas.mrcieu.ac.uk/)
Ebi‐a‐GCST004884	(https://gwas.mrcieu.ac.uk/)
Ebi‐a‐GCST004885	(https://gwas.mrcieu.ac.uk/)
Ebi‐a‐GCST90001482	(https://gwas.mrcieu.ac.uk/)
Ebi‐a‐GCST90001481	(https://gwas.mrcieu.ac.uk/)
418/211 gut microbiota	(https://gwas.mrcieu.ac.uk/)
412 gut microbiotas	(https://www.ebi.ac.uk/gwas/publications/35115690)
Blood cell	(http://www.mhi‐humangenetics.org/en/resources/)
91 inflammation‐related proteins	(https://www.ebi.ac.uk/gwas/publications/37563310)
41 inflammatory cytokines	(https://www.bristol.ac.uk/)
Metabolites	(https://gwas.mrcieu.ac.uk/)
Mitochondrial	(https://gwas.mrcieu.ac.uk/)
Serum metabolites	(https://www.ebi.ac.uk/gwas/publications/36635386)
Immune cell default	(https://www.ebi.ac.uk/gwas/publications/32929287)
Immune cell original	(https://gwas.mrcieu.ac.uk/)
Lipidome	(https://www.ebi.ac.uk/gwas/publications/37907536)
Skin PopGen/KORA	(https://www.ebi.ac.uk/gwas/publications/36261456)
TARGET	(https://ocg.cancer.gov/programs/target)

### 2.2 Determine instrumental variables

Based on the Mendelian laws of genetics, we use the association between known SNPs and a specific trait or disease to randomly group individuals according to their genotype. The purpose of this random grouping is to simulate the impact of randomization on NB, to infer the causal relationship between this SNP and NB, and due to the lack of clear elucidation of the pathogenesis of NB, we organized the common exposure factors mentioned above and screened IVs in the five datasets of NB. For the initial exposure and outcome, we used three models: inverse variance weighted (IVW), Weighted Median, and MR Egger for analysis. *p* < 5 × 10^−8^ was used as the significance threshold for the whole genome (as some variables were rarely identified as SNPs during exposure, higher cutoff values were selected, *p* < 5 × 10^−6^) to screen SNPs with a strong correlation between exposure and NB in each exposure dataset. We used the forest plot R package to draw the final forest map. With the help of website (http://jvenn.toulouse.inra.fr/), we drew a Venn diagram and analyzed the selected relevant exposures.

### 2.3 Two sample Mendelian randomization

To evaluate the accuracy of the potential causal relationship between NB and the selected variables (percentage of CD4 regulated T cells in resting state), we searched the IEU Open GWAS website for datasets "ebi‐a‐GCST90001481" and "ebi‐a‐GCST90001482" corresponding to Resting CD4 regulated T cells as exposures and selected the NB dataset "prot‐a‐2003" with the largest sample size as the outcome. Two sample MR analyses were conducted using the "two sample MR" R package (version 3.6.3), IVW, Weighted median, and MR Egger models were used. We conducted a sensitivity analysis using the "MR presso" R package (version 3.6.3) to evaluate the level of validity of the model.

### 2.4 Co‐localization analyses

Afterward, we identified genetic variation sites related to phenotype based on GWAS results using quantitative trait loci (QTL) loci published in existing databases.^24^ We conducted co‐localization analysis to identify whether the NB phenotype and corresponding inflammatory cytokine phenotype are driven by the same causal variation in a certain region in order to confirm the evidence of a correlation between the two phenotypes. We used the "Coloc" R package to perform co‐localization analysis. Subsequently, we searched for corresponding expression quantitative trait loci (eQTL), protein quantitative trait loci (pQTL), and metabolite quantitative trait loci (mQTL) for the "ebi‐a‐GCST90001481" and "ebi‐a‐GCST90001482" datasets. Finally, we retrieve the SNP loci from the gene tracks in the "MR instrument" R package and search for the genes corresponding to the coordinate positions. We use the Therapeutically Applicable Research to Generate Effective Treatments (TARGET, *n* = 159) database to determine the feasibility of the genes.

### 2.5 Summary‐data‐based Mendelian randomization

SMR tests the pleiotropic association between gene expression levels and NB complex traits by combining GWAS and QTL.^25^ Because we only found IVs related to the percentage of CD4 regulated T cells in the resting state from "prot‐a‐2003" and "ebi‐a‐GCST004883", in order to further explore genes related to NB, we used eQTL (GTEx V8) as the underlying data. From "prot‐a‐2003" and "ebi‐a‐GCST004883", we selected "Genome Assembly Gold‐Standard Evaluations (GAGE)" and "adrenal" as conditions and screened them according to a *p* < 5 × 10^−4^. Finally, the two results were combined to obtain more comprehensive results. The HEterogeneity In Dependent Instruments (HEIDI) *p* < 0.05 was attributed to the presence of a correlation between genes. Due to pleiotropy, we excluded them and defined the NB set composed of remaining genes for subsequent analysis. Prior to this, we used the web tool (https://cn.string‐db.org/) to analyze the interactions between UBXN6 proteins and excluded (HEIDI *p* < 0.05) genes' proteins.

### 2.6 Drug‐screening

Finally, based on the NB set mentioned above, we used network pharmacology proximity algorithms for drug prediction and obtain the target genes for the corresponding drugs from the drug database (https://go.drugbank.com/). Next, we calculated the shortest path between the drug target genes and the NB set. If the distance between the drug target genes and the NB set is closer, it indicates that the drug has a certain effect on the treatment of NB. The distance between the drug and the NB set is called “proximity”, which is converted into a z‐score. After 1000 random perturbations, the significant *p*‐value of each drug can be calculated to screen for targeted drugs,^26^ and then we obtain the corresponding 3D structure of the drug from the website (http://drugmap.idrblab.net/).

### 2.7 Statistic analyses

The IVW method is the main method for MR analysis. When the effective IVs exceed 50%, the weighted median method can obtain a consistent estimate of the overall effect and reduce bias in causal effect estimation. Compared to the IVW method, the MR Egger method is used to determine whether instrumental SNP has pleiotropy. The Cochrane Q‐statistics of IVW were used to measure the heterogeneity between each SNP estimate. In addition, sensitivity analysis also used simple mode analysis and weighted mode analysis. For the screened variables, we used Bonferroni correction to calculate *p* < 0.05 (the result of the screened exposure×outcome or exposure×screened outcome), which means that the association result is *p* < 0.05 before multiple comparison correction, rather than a significant result of *p*‐value after multiple comparison correction. The *p*‐value is higher than the Bonferroni correction threshold but lower than 0.05, which is considered as implicit evidence of potential causal relationships. All statistical analyses are bilateral analyses.

## 3 RESULTS

### 3.1 Immune cell

**FIGURE 1:**
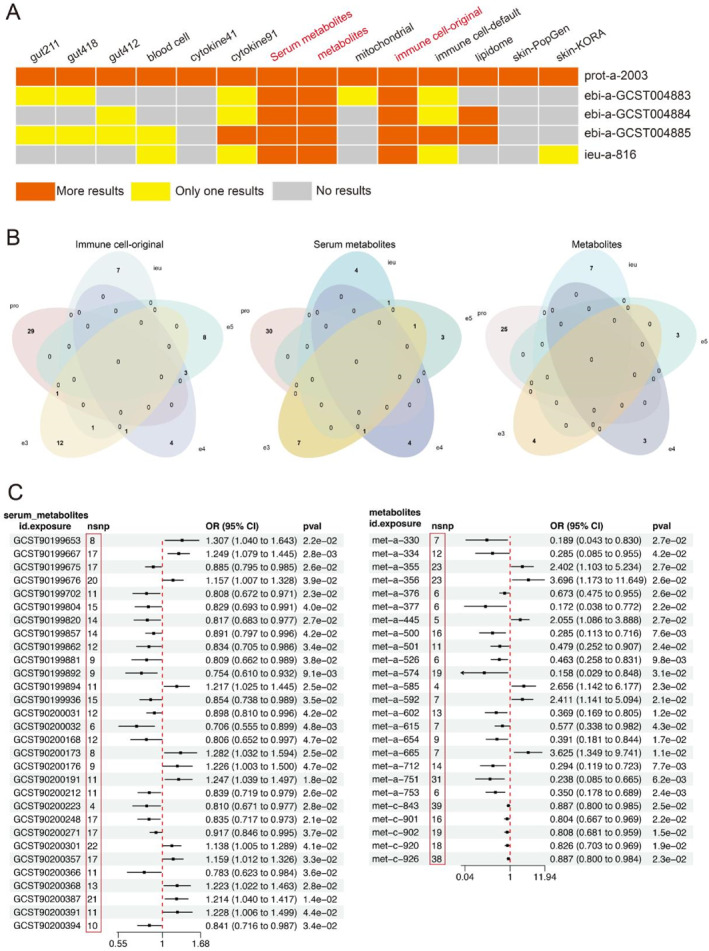
Preliminary screening (A) Summary of results from various exposure screening, exposure includes intestinal flora, blood cell, inflammatory factors, metabolites, serum metabolites, mitochondrial related variables, immune cells, liposomes, and skin flora; (B) Venn diagram of three exposed datasets on five NB outcome datasets, exposure includes immune cells, metabolites and serum metabolites; (C) Forest map of exposure metabolites and serum metabolites screening in outcome prot‐a‐2003.

In the exposure screening of the five outcome NB datasets, we summarized all screening results. In a total of five datasets consisting of 91 cell inflammatory factors, metabolites, serum metabolites, and immune cells, all relevant exposures were screened. However, in the two exposure datasets consisting of 91 cell inflammatory factors and default immune cells, we only screened a single exposure in the three outcome NB datasets of "ebi‐a‐GCST004883", "ebi‐a‐GCST004884", and "ieu‐a‐816"(Figure 1A). This may be due to the bias caused by the small number of SNPs in the outcome NB dataset. However, in order to ensure the results are more reliable, we excluded these two exposure datasets (91 cell inflammatory factors and default immune cells) and chose three exposure datasets: metabolites, serum metabolites, and original immune cells for further analysis.

Since the "prot‐a‐2003" dataset had the highest number of SNPs among the 5 outcome NB datasets, the results obtained were more universal. Therefore, in the screening results of the metabolite, serum metabolite, and original immune cell exposure datasets, we only selected the exposure variables that intersected with "prot‐a‐2003", and the remaining intersection variables were eliminated. Finally, in the analysis of the original immune cell exposure dataset, we included the intersection result of "prot‐a‐2003" and "ebi‐a‐GCST004883" in the "Resting CD4 regulatory T cell% CD4 + T cell (ebi‐a‐GCST90001482) as the follow‐up research object (Figure 1B).

### 3.2 Resting CD4 regulatory T cell %CD4 + T cell

**FIGURE 2:**
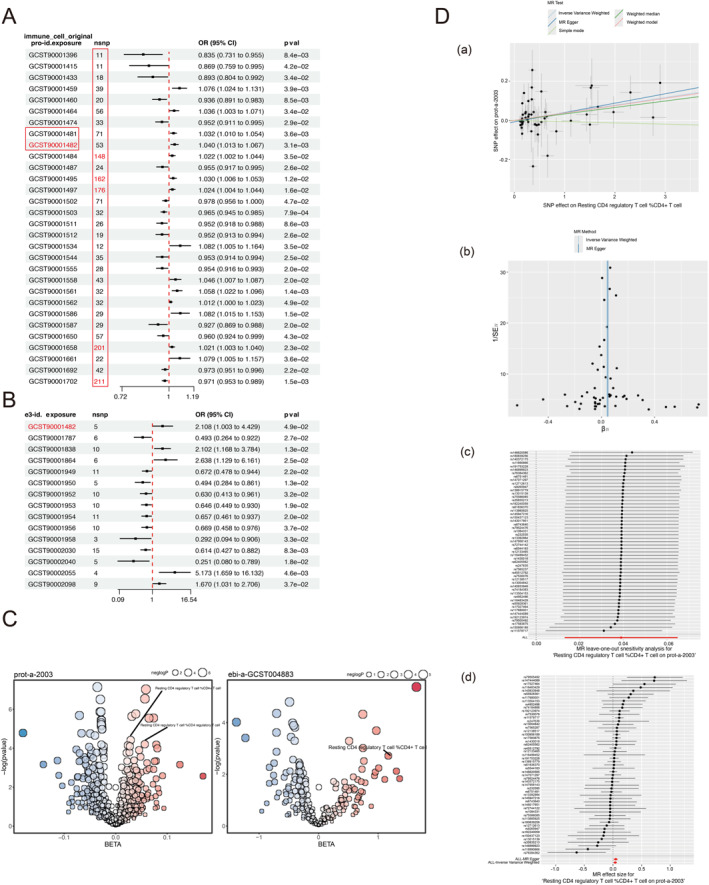
Identifying exposure testing CD4 regulatory T cell %CD4 + T cell (A) forest map of exposure immune cell original screening in outcome prot‐a‐2003; (B) Forest map of exposure immune cell original screening in outcome ebi‐a‐GCST004883; (C) Bubble‐plot of exposure resting CD4 regulatory T cell %CD4 + T cell in outcome prot‐a‐2003 and ebi‐a‐ GCST004883, resting CD4 regulatory T cell %CD4 + T cell includes GCST90001481 and GCST90001482; (D) NB and resting CD4 regulatory T cell %CD4 + T cell, two sample Mendelian randomization (MR) analysis. a: each point corresponds to an SNP site, with the horizontal axis representing the effect of SNP on the exposure variable resting CD4 regulatory T cell %CD4 + T cell and the vertical axis representing the effect of SNP on the outcome variable (NB). The lines represent the MR fitting results. b: the funnel plot shows the inverse variance weighted (IVW) MR estimation and 1/standard error (SE) of each inflammatory factor SNP and NB. c: leave one out snesitivity analysis. d: forest diagram of SNP.

The number of SNPs in the screening results of "resting CD4 regulatory T cell% CD4 + T cell (ebi‐a‐GCST90001482)" in "prot‐a‐2003" was 53. Although it was not the highest number of SNPs in the screening results of "prot‐a‐2003" and original immune cell screening, it was also higher than the maximum number of SNPs corresponding to the results obtained in the exposure datasets of metabolites and serum metabolites in "prot‐a‐2003". The maximum number of SNPs in the screening results of serum metabolites in "prot‐a‐2003" was 22, and the maximum number of SNPs in the screening results of metabolites was 39, both of which were less than 53. Moreover, the overall number of SNPs corresponding to exposure obtained by "prot‐a‐2003" in the original immune cell dataset was higher than that obtained by "prot‐a‐2003" in the serum metabolite and metabolite datasets (Figure 1C, Figure 2A). This may be due to the stronger correlation between the features of the original immune cell and the NB suppressor of tumorigenicity 1(prot‐a‐2003).

### 3.3 Resting CD4 regulatory T cell %CD4 + T cell and NB

As an exposure, "resting CD4 regulatory T cell% CD4 + T cell (ebi‐a‐GCST90001482)" showed consistency in both "prot‐a‐2003" and "ebi‐a‐GCST004883" NB outcomes (Figure 2A,B), indicating that with an increase in resting CD4 regulatory T cell% CD4 + T cell, the likelihood of NB occurrence and development increased. Moreover, the exposure variable "ebi‐a‐GCST90001481", which has the same characteristics as "ebi‐a‐GCST90001482" (resting CD4 regulatory T cell% CD4 + T cell), showed consistency in the results of "prot‐a‐2003"(Figure 2A,C), which further confirms our conclusion. Although "ebi‐a‐GCST004883" NB outcome was not located in the result of the exposure variable "ebi‐a‐GCST90001481", this may be due to the larger number of SNPs contained in the "prot‐a‐2003" dataset.

Later, in the analysis of the two sample MR of "ebi‐a‐GCST90001482" and "prot‐a‐2003", we observed a positive correlation between the increase in resting CD4 regulatory T cell% CD4 + T cell and the occurrence of NB (Figure 2Da). When the SNP effect on resting CD4 regulatory T cell %CD4 + T cell approaches 0, the outcome variable (effect on prot‐a‐2003) almost approaches 0, which indicates almost no multiple effects (confounding factors). Although the Simple mode *p* = 0.829, Weighted median *p* = 0.079, MR Egger *p* = 0.05, Weighted Mode *p* = 0.021, the most critical IVW mode *p* = 0.003, the Weighted mode, and the IVW mode have consistent b‐value directions. The funnel plot shows a symmetrical shape (Figure 2Db), and no clear heterogeneity was found in the heterogeneity test (MR Egger *p* = 0.259; IVW *p* = 0.263). The horizontal pleiotropy test value is *p* = 0.373, so it can be ignored. The exposure (resting CD4 regulatory T cell% CD4 + T cell) appears upstream of the outcome(NB) (*p < *0.001).

Using the leave one out sensitivity analysis (Figure 2Dc), we determine whether a certain SNP has significantly changed the results by removing SNPs one by one. ALL>0 indicates that the results are credible. Each horizontal solid line in the forest map represents the result estimated using the Wald ratio method based on a single SNP(Figure 2Dd): those completely to the left of 0 indicate that an increase in resting CD4 regulatory T cell% CD4 + T cell will reduce NB risk; completely to the right of 0 indicates that an increase in Resting CD4 regulatory T cell% CD4 + T cell will increase NB risk; crossing 0 indicates that the result is not significant, therefore, All Inverse Variance Weighted>0 and All Mendelian Randomization Egger>0 indicate that our result is credible.

### 3.4 Related genes of resting CD4 regulatory T cell %CD4 + T cell

**FIGURE 3:**
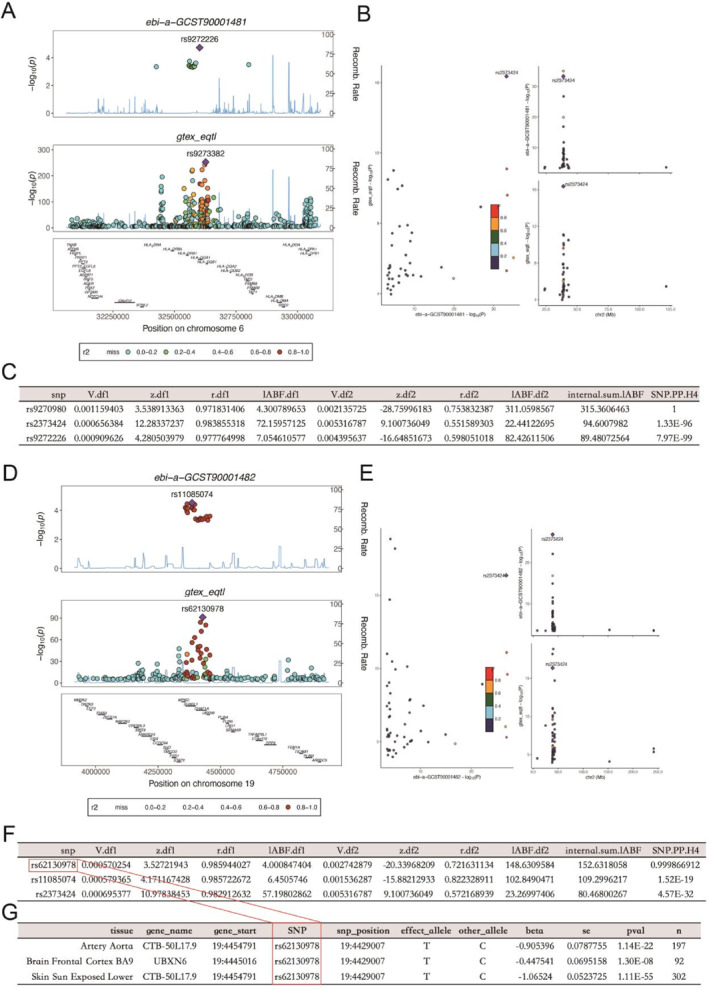
Identifying genes: (A) Based on GCST90001481 and GWAS‐eQTL, search for common rs sites between them; (B) Scatter plot displays the corresponding lead SNP; (C) Co‐localization analysis results, rs9270980‐H4>0.75; (D) Based on GCST90001482 and GWAS‐eQTL, search for common rs sites between them; (E) Scatter plot displays the corresponding lead SNP; (F) Co‐localization analysis results, rs62130978‐*H*4>0.75; (G) Corresponding genes in gene trails.

We observed corresponding loci rs9272226 and rs9273382 in GWAS‐eQTL from the "ebi‐a‐GCST90001481" dataset, with the Lead SNP locus being rs2373424. Unfortunately, the rs9270980 locus with *H* ^4^>0.75 did not mediate the related phenotype in GWAS (Figure 3A/B/C). The "ebi‐a‐GCST90001482" dataset observed corresponding loci rs11085074 and rs62130978 in GWAS‐eQTL, with the lead SNP locus being rs2373424 and the *H* ^4^>0.75 locus being rs62130978 corresponding to the genes *UBXN6* and *CTB‐50L17.9* in gene trails. With the help of the TARGET database, the *CTB‐50L17.9* gene that was not found in the database was excluded. We ultimately chose *UBXN6* as the gene corresponding to the rs62130978 locus, which also indicated *UBXN6* corresponding to the resting CD4 regulatory T cell %CD4 + T cell. We found that this gene is mainly located in brain frontal cortex BA9 expression (Figure 3D,E,F,G). However, we did not find any relevant SNP sites in the GWAS datasets of pQTL and mQTL, which may be due to the insufficient number of SNPs in "ebi‐a‐GCST90001481" and "ebi‐a‐GCST90001482".

### 3.5 Related genes of NB

**FIGURE 4:**
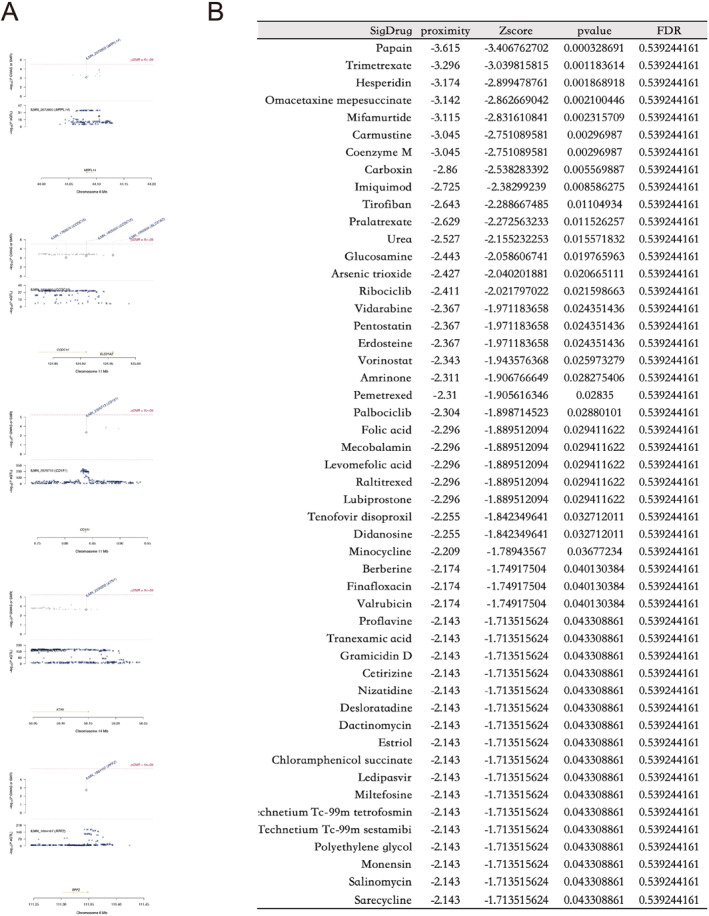
Summary‐data‐based Mendelian randomization (SMR) and drug. (A) Among the genes screened by SMR in the "GAGE" module of "prot‐a‐2003", genes with HEIDI‐*P*<0.05 include *CCDC15*, *KTN1*, *MRPL14*, *RPF2*, and *CD151*; (B) The top 50 drugs screened by the network pharmacology proximity algorithm; list are arranged in ascending order of *p*. GAGE. Genome Assembly Gold‐Standard Evaluations; HEIDI, HEterogeneity In Depedent Instruments.

In the SMR screening of the "GAGE" module in "prot‐a‐2003", five genes with HEIDI‐*P* < 0.05 were identified, including *CCDC15*, *KTN1*, *MRPL14*, *RPF2*, and *CD151*. *MRPL14* and *RPF2* are located on chromosome 6; *CCDC15* and *CD151* are located on chromosome 11, and their adjacent gene *SLC37A2* was observed in *CCDC15*; *KTN1* is located on chromosome 14, and its adjacent gene locus was not found (Figure 4A). However, the genes screened by SMR in the "adrenal" module of "prot‐a‐2003" did not contain genes with HEIDI‐*P* < 0.05. The "GAGE" and "adrenal" modules of ebi‐a‐GCST004883 also do not contain genes with HEIDI‐*P* < 0.05. After excluding five genes, *CCDC15*, *KTN1*, *MRPL14*, *RPF2*, and *CD151*, we conducted network pharmacology analysis using the remaining gene composition set (NB set). Although the proteins corresponding to these gene sets do not have strong interactions with the *UBXN6* protein, this may be due to the small sample size of our dataset.

### 3.6 Target drug of NB

**FIGURE 5:**
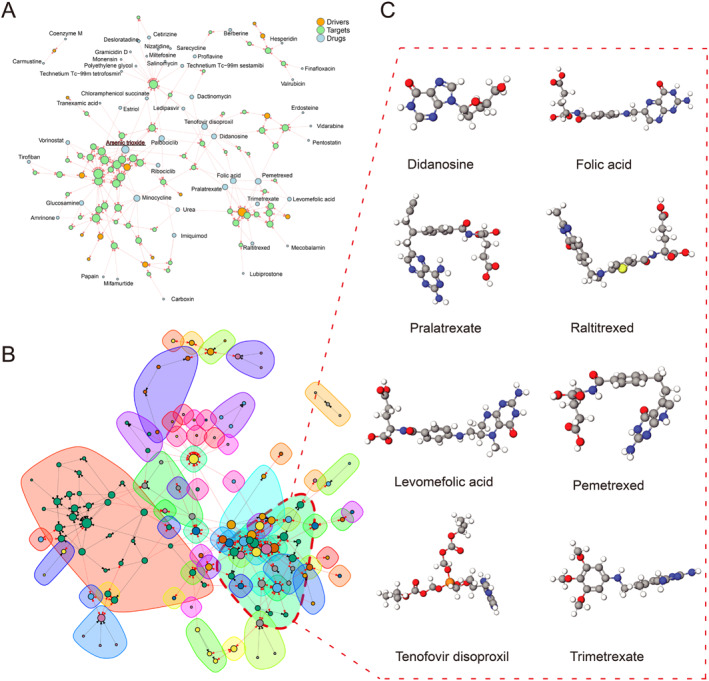
Drug Analysis: (A) The interaction network composed of driving genes, target genes, and target drugs in the top 50 drugs; (B) Interaction network clustering; (C) The 3D structure of drugs didanosine, tenofovir dioproxil, levomefolic acid, pralatrexate, trimetrexate, pemetrexed, raltitrexed, and folic acid.

Using a gene set (NB set) consisting of 124 genes as the underlying data, we utilized network pharmacology proximity to screen for relevant targeted drugs. Figure 4B shows the top 50 drugs with significant *p*‐value, among which the drug papain has the strongest characteristic. From the interaction network constructed by the 50 drugs, we found that apart from the drugs coenzyme M and carmustine, the other 48 drugs and targets formed an overall interconnected network, while the target of the drug arsenic trioxide was the densest (Figure 5A). Subsequently, we clustered these drugs and discovered that didanosine, tenofovir disoproxil, levomefolic acid, pralatrexate, trimerexate, urea, pemetrexed, raltitrexed, folic acid, and mecobalamin constitute the largest drug subgroup (Figure 5B), while the remaining subgroups contain too few drugs. Due to the website's limitations (http://drugmap.idrblab.net/), we did not find the 3D structures corresponding to mecobalamin and urea in the dataset, so we visualized the 3D structures of eight drugs: didanosine, tenofovir dioproxil, levomefolic acid, pralatrexate, trimetrexate, pemetrexed, raltitrexed, and folic acid (Figure 5C).

## 4 DISCUSSION

In this study, we integrated the current common exposure factor datasets and used all the NB datasets from IEU Open GWAS as the outcome. We found that resting CD4 regulatory T cell% CD4 + T cell was positively correlated with the occurrence and development of NB. Then, we combined co‐localization analysis and SMR to determine NB set of genes, providing drug guidance for NB chemotherapy. This study, as the first to integrate all GWAS public databases to explore the risk factors of NB, systematically screened the risk factors of NB and improved our understanding of the molecular mechanisms associated with the onset of NB.

At first, when we screened for exposure factors, it was difficult to locate the relevant exposure in the gut microbiota, blood cells, mitochondria, and skin microbiota. And this is also related to the relatively small number of SNPs in the database we have collected, the result is reasonable. NB, as a cancer developed from immature sympathetic nerve cells, has less association with above exposure. On the contrary, we have found many related exposures in the dataset of immune cells, metabolites, and serum metabolites: the relationship between immune cells and tumors has always been a "hot topic" in the research field. Whether it is the exploration of "tumor microenvironment" or "immune microenvironment", the relationship between immune cells and tumors is complex and interdependent. NB is formed by abnormal cell proliferation in the body, and in this process of abnormal cell proliferation, it is inevitable that the metabolic rate of cells will accelerate and the metabolic products will increase. Therefore, it is reasonable to find many NB related exposures in the metabolite related data set. Finally, we determined a positive correlation between "resting CD4 regulatory T cell% CD4 + T cell" and the occurrence and development of NB, that is, as the resting CD4 regulatory T cell% CD4 + T cell increases, the risk of NB increases. T cell vaccine induces CD4 regulatory T cell response in patients with multiple sclerosis,^27^ however, there are currently no reports on CD4 regulatory T cells and NB.

Afterward, we attempted to use co‐localization analysis to identify relevant genes from the resting CD4 regulatory T cell% CD4 + T cell. We identified genetic variation sites related to phenotype based on GWAS results using QTL locally published in existing databases, and ultimately identified the rs62130978 site as a site related to the resting CD4 regulatory T cell% CD4 + T cell phenotype, with corresponding genes *UBXN6* and *CTB‐50L17.9* defined. Because the TARGET database contains a comprehensive range of genes in NB's transcriptome database, we use it as a reference. *CTB‐50L17.9* was not found in the TARGET database, so we excluded it. And the main expression sites of *CTB‐50L17.9* are artery aorta and skin sun exposed lower, which are not directly related to NB, and there have been no reports of metastasis to these sites in NB. *UBXN6* is mainly expressed in brain frontal cortex BA9, and there have been reports of NB metastasis in the brain.^28^ Although SNP of *UBXN6* is associated with long term non progression phenotype in Human Immunodeficiency Virus positive individuals, there are currently no reports on the association between *UBXN6* and NB.^29^

In addition, we constructed gene sets from the SMR results of the two databases, "prot‐a‐2003" and "ebi‐a‐GCST004883", which screened out "resting CD4 regulatory T cell% CD4 + T cell". The results showed that the *p*‐value in the HEIDI test of *CCDC15*, *KTN1*, *MRPL14*, *RPF2*, and *CD151* was less than 0.05, indicating that the correlation of these genes may be caused by pleiotropy. *CCDC15* is located within the scaffold of the centriole and controls the length and integrity of the centriole.^30^ *KTN1*, as an LncRNA, promotes tumor growth in hepatocellular carcinoma and exacerbates tumor progression in ovarian cancer.^31^, ^32^ *MRPL14* not only promotes the biogenesis and mitochondrial translation of mitochondrial macroribosomal subunits, but also has certain relevance in diabetes retinopathy.^33^, ^34^ *RPF2* can promote chemotherapy resistance in colorectal cancer cells.^35^ There are many reports on *CD151* and tumors, which not only play a core role in tumor progression, but also play a significant role in the immune microenvironment of tumors. Specifically, it can inhibit the movement of prostate cancer tumor cells during aggregation and regulate tumor occurrence by regulating communication between tumor cells and endothelial cells.^36^, ^37^, ^38^, ^39^ These genetic variations not only affect the specific phenotype of the GAGE module, but may also affect other phenotypes through pathways independent of exposure factors.

Finally, we constructed a gene set from the SMR results of two databases, "prot‐a‐2003" and "ebi‐a‐GCST004883". We used network pharmacology proximity algorithms to screen for NB targeted drugs, among that didanosine, tenofovir disoprostil, levomefolic acid, pralatrexate, trimerexate, urea, pemetrexed, raltitrexed, folic acid, and mecobalamin represent a large class of drugs because their target and driver gene positions are similar, but the drug with the most significant *p*‐value is still papain. Studies have shown that targeting papain can inhibit broad‐spectrum coronavirus, and papain also exhibits the kinetic behavior of chitosan degradation. When the initial concentration of chitosan is higher than 8.0 g/L, it is overloaded and exhibits significant inhibitory effects. After papain treatment, it will have an impact on the aggregation of Novikoff tumor cells.^40^, ^41^, ^42^ Moreover, after B cell receptor, independent papain uptakes, B cells regulate the response of CD4 + T cells to papain.^43^ This further supports that the drug papain can affect NB by regulating the resting CD4 regulatory T cell% CD4 + T cell.

## 5 CONCLUSION

The increase of resting CD4 regulatory T cell is positively correlated with the occurrence and development of NB, while the drug papain targets CD4 + T lymphocytes and has a chemotherapy effect on NB.

## 6 LIMITATIONS

Although the study yielded satisfactory results and inferences, further validation is still needed through in vivo and in vitro experiments, we also can explore and search for more comprehensive NB databases on other platforms for similar analysis to validate our conclusions.

## AUTHOR CONTRIBUTIONS

RuiZong Wang was responsible for the overall conception and design of the study. Shan Wang revising the manuscript.

## CONFLICT OF INTEREST STATEMENT

The authors declare that they do not have any conflict of interests.

## ETHICS STATEMENT

Since this study only uses publicly available data, there is no need to obtain additional ethical approval from the institutional review committee. The research performed under the conformity to the principles of the Helsinki Declaration.

## Data Availability

The data we use is based on published research and public databases and the data used in the study is summarized in a Table 1.
